# In-depth analysis of *Klebsiella aerogenes* resistome, virulome and plasmidome worldwide

**DOI:** 10.1038/s41598-024-57245-1

**Published:** 2024-03-19

**Authors:** Sergio Morgado, Érica Fonseca, Fernanda Freitas, Raquel Caldart, Ana Carolina Vicente

**Affiliations:** 1 Laboratório de Genética Molecular de Microrganismos, Instituto Oswaldo Cruz, Rio de Janeiro, RJ 21040-360 Brazil; 2 Centro de Ciências da Saúde, Universidade Federal de Roraima, Boa Vista, RR 69300-000 Brazil

**Keywords:** Computational biology and bioinformatics, Genetics, Microbiology

## Abstract

*Klebsiella aerogenes* is an emergent pathogen associated with outbreaks of carbapenem-resistant strains. To date, studies focusing on *K. aerogenes* have been small-scale and/or geographically restricted. Here, we analyzed the epidemiology, resistome, virulome, and plasmidome of this species based on 561 genomes, spanning all continents. Furthermore, we sequenced four new strains from Brazil (mostly from the Amazon region). Dozens of STs occur worldwide, but the pandemic clones ST93 and ST4 have prevailed in several countries. Almost all genomes were clinical, however, most of them did not carry ESBL or carbapenemases, instead, they carried chromosomal alterations (*omp*36, *amp*D, *amp*G, *amp*R) associated with resistance to β-lactams. Integrons were also identified, presenting gene cassettes not yet reported in this species (*bla*IMP, *bla*VIM, *bla*GES). Considering the virulence loci, the yersiniabactin and colibactin operons were found in the ICEKp10 element, which is disseminated in genomes of several STs, as well as an incomplete salmochelin cluster. In contrast, the aerobactin hypervirulence trait was observed only in one ST432 genome. Plasmids were common, mainly from the ColRNAI replicon, with some carrying resistance genes (*mcr*, *bla*TEM, *bla*NDM, *bla*IMP, *bla*KPC, *bla*VIM) and virulence genes (EAST1, *sen*B). Interestingly, 172 genomes of different STs presented putative plasmids containing the colicin gene.

## Introduction

The bacteria formerly known as *Enterobacter aerogenes* has recently been renamed *Klebsiella aerogenes* based on whole-genome sequence phylogenetics^1,2^. This species is a ubiquitous member of the *Enterobacteriaceae* family, and although it has always been considered an opportunistic pathogen, recently, interest in this organism has increased, mainly due to the emergence of multidrug-resistant and carbapenem-resistant strains^3^. This may be due to the acquisition of antibiotic resistance genes carried by mobile elements, such as plasmids and conjugative integrative elements, which have already been identified in *K. aerogenes*^4–6^. However, in this species, the main mechanisms of resistance to carbapenems are attributed to chromosomal overexpression of AmpC β-lactamase (e.g., *bla*_DHA-1_ and *bla*_CMY-2_) and mutations that affect membrane permeability^4,7,8^. Furthermore, reports show that polymyxin resistance is occurring in strains of this species due to mutations in some housekeeping genes (*mgr*B, *pmr*A, *pmr*B, and *ept*A)^9,10^ and, eventually, by plasmids carrying the *mcr* gene.

There are continuous reports of the high frequency of outbreaks of this emergent pathogen, some of them associated with high mortality rates^11,12^, in different clinical settings around the world, such as neonatal^13–15^, geriatric^16^, and intensive care units^8,17^. Thus, *K. aerogenes* was included in the ESKAPE group, which encompasses important pathogens associated with antimicrobial resistance^5^. Among the known *K. aerogenes* lineages, comparative genomics showed that type 4 (ST4) and ST93 lineages likely represent the dominant lineages associated with human infections worldwide^4^. In addition, some lineages have been associated with virulence determinants, such as siderophores and toxins, which potentiate their persistence and favor the emergence of nosocomial outbreaks^4,5,10,14^.

So far, studies focusing on *K. aerogenes* have been small-scale and/or with a restricted geographic perspective^4,5,18^. Here, in addition to generating new genomes of this species in Brazil and contextualizing them in the global scenario, we performed an in-depth analysis of the resistome, virulome, and plasmidome of this emergent pathogen.

## Results

### *K. aerogenes* sequence typing and epidemiology

In this study, we analyzed four strains of *K. aerogenes* from nosocomial cases from the Amazon and Southeast Brazil: Ka-01RR (subclavian vein catheter tip), Ka-02RR (tracheal secretion), Ka-04RR (unknown), Ka-06RJ (sputum). These strains were subjected to whole genome sequencing using the Illumina platform. The assembly statistics of these four genomes are presented in Table 1. The size of the genomes ranged from 5,043,146 to 5,175,129 bp with a GC content of ~ 55% and encoding 4728 to 4815 protein-coding sequences (CDS), 7–9 rRNAs, and 78–80 tRNAs (Table 1). Although the coverage depth of the genomes is not as high as commonly in Illumina runs, all genomes had size and number of CDS compatible with the species, as well as high genomic completeness (BUSCO > 95%).

**Table 1:** Assembly information of sequenced Brazilian genomes.

	Complete BUSCO (%)	Coverage depth	# Contigs	Largest contig (bp)	Total length (bp)	N50	L50	GC (%)	CDS	rRNA	tRNA
Ka-01RR	95.95	19	391	85,021	5,045,871	22,918	65	55.12	4758	7	79
Ka-02RR	95.95	23	364	97,818	5,043,146	25,629	62	55.12	4728	8	78
Ka-04RR	95.95	23	373	93,200	5,175,129	25,357	61	55.06	4815	7	78
Ka-06RJ	97.3	24	238	319,657	5,097,888	41,592	33	55.03	4752	9	80

**Figure 1:**
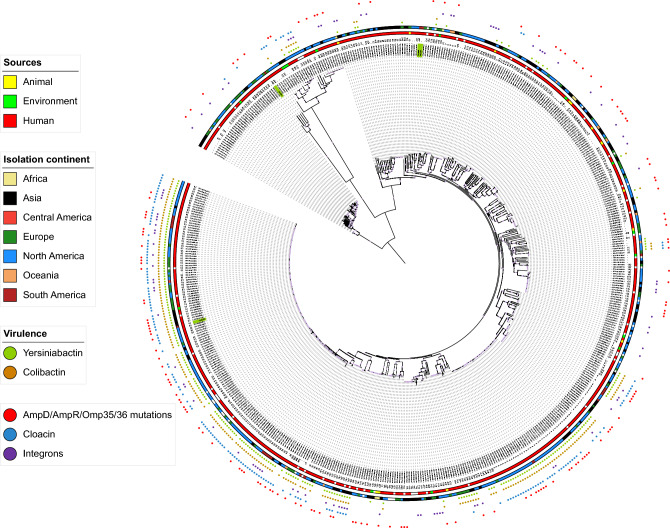
Maximum-likelihood tree based on the core gene alignment of the 561 K*. aerogenes* genomes. The ST number of each genome is next to the accession number. There are two orbits of colored blocks, where the innermost represents the source of isolation, and the outermost represents the regions of the genomes. Next, five colored circles indicate genomes with yersiniabactin (green), colibactin (beige), integron (purple), cloacin (blue), nonsense mutations in AmpD, AmpR, Omp35/36 (red)^4,20,21^. The genomes sequenced here are in green background color. Red circles at branches indicate bootstrap values > 80%.

In total, 561 *K aerogenes* were analyzed, where most (n = 477) were associated with human infections, and others were associated with the environment (n = 15) and animals (n = 13), covering the period from 1955 to 2022. These genomes were spread across 37 countries, with a bias towards the US, which corresponds to 44% (n = 247) of the genomes in the dataset. Only eight countries have more than a dozen genomes (Australia, Germany, Brazil, Canada, UK, China, Singapore, USA) (Table S1). For all sources, several STs were identified, where ST93 (n = 121) and ST4 (n = 57) were the most prevalent (Table S1). Other STs were present in no more than 20 genomes, showing that the prevalence of ST93 and ST4 is indeed much higher (Table S1). A maximum likelihood tree was constructed based on the core genes (n = 2319) of the 561 genomes (Fig. 1) and showed the division of the genomes into four main clusters, with the main one encompassing most of the genomes (493/561) into several subclusters, including ST4 and ST93 (Fig. 1). 41 genomes did not have any ST assigned, showing the genetic diversity of this species. Therefore, we submitted these allelic patterns to pubMLST database (https://pubmlst.org/bigsdb?db=pubmlst_kaerogenes_seqdef), where 41 new ST profiles were generated and included in this study (ST398-ST437, and ST440). The ST4 genomes are from 2004 to 2022 and occur in several countries in South America, North America, Asia, and, only in the United Kingdom in Europe. Most of this ST is associated with human sources; however, one genome is from the environment (Table S1). ST93 is more widespread than ST4 genomes as it also occurs in several European countries and Central America. Most ST93 genomes are from humans, occurring from 1997 to 2022 (Table S1).

In Brazil, 16 *K aerogenes* genomes were associated with human infections from 2006 to 2016 in three states in the Southeast region (Minas Gerais, Paraná and São Paulo), where three STs were observed: ST93 (11/20), ST16 (2 /20) and ST4 (3/20) (Table S1). Furthermore, the four new genomes sequenced here came from other states in the Southeast region, Rio de Janeiro (ST296), and from the North region, Roraima (ST93 and ST440). ST296 and ST440 represent two new STs in Brazil.

### Brazilian *K. aerogenes* antibiotic susceptibility

The four Brazilian strains of *K. aerogenes* were tested for various antibiotics, including carbapenems and cephalosporins (Table 2). The Ka-04RR strain showed resistance to a greater number of antibiotics, mainly cephalosporins and β-lactams. The punctual pattern of resistance to carbapenems, cephalosporins and β-lactams suggests the absence of an enzymatic resistance mechanism. Even so, all strains would be considered multidrug resistant (MDR)^19^.

**Table 2:** Antibiotic susceptibility of Brazilian *K. aerogenes.*

Antibiotic classes	Antibiotics	Strains			
		Ka-01RR	Ka-02RR	Ka-04RR	Ka-06RJ
Aminoglycosides	AMK	S	I	I	S
	GEN	S	I	S	S
	STR	S	I	R	S
	TOB	S	R	S	S
	NEO	S	I	S	S
	KAN	S	I	S	I
β-Lactams: carbapenems	IPM	R	I	S	S
	MEM	I	I	R	R
	ERT	S	S	S	S
β-Lactams: cephalosporins	CAZ	R	S	R	S
	CRO	S	S	R	S
	CTX	I	I	R	S
	FEP	I	S	I	S
	FOX	S	S	R	R
	CXM	I	S	R	S
β-Lactams: others	SAM	R	I	R	S
	TZP	S	S	R	S
	TIM	S	S	R	S
Quinolones	CIP	I	R	I	I
	LEV	S	I	S	S
Sulfonamides/antifolate	SXT	S	R	S	S
Tetracyclines	MIN	R	R	S	S
	TET	S	R	S	S
Polymyxins	COL	S	S	S	S

*AMK* amikacin, *GEN* gentamicin, *STR* streptomycin, *TOB* tobramycin, *NEO* neomycin, *KAN* kanamycin, *IPM* imipenem, *MEM* meropenem, *ERT* ertapenem, *CAZ* ceftazidime, *CRO* ceftriaxone, *CTX* cefotaxime, *FEP* cefepime, *FOX* cefoxitin, *CXM* cefuroxime, *SAM* ampicillin/sulbactam, *TZP* piperacillin/tazobactam, *TIM* ticarcillin/clavulanic acid, *CIP* ciprofloxacin, *LEV* levofloxacin, *SXT* trimethoprim/sulfamethoxazole, *MIN* minocycline, *TET* tetracycline, *COL* colistin. Resistance profile: *S* susceptible, *R* resistant, *I* intermediate.

### *K. aerogenes* resistome analysis

The resistome of *K. aerogenes* genomes is heterogeneous, with 245 genomes presenting several genes associated with resistance to aminoglycosides, quinolones, macrolides, phenicol, sulfonamide, trimethoprim and β-lactams, while we did not observe acquired resistance genes in 316 genomes (Table S1). Among the β-lactamases genes, only 42 and 150 genomes, respectively, presented extended-spectrum β-lactamases (ESBL) and carbapenemases genes (Table S1). Therefore, although most genomes are of clinical origin, most of them do not carry carbapenemases. The most prevalent carbapenemases were *bla*_KPC_ (− 2 and − 3), *bla*_OXA-48_ and *bla*_NDM_ (− 1, − 5, − 7 and − 9) (Table S1). Most *bla*_KPC_ and *bla*_NDM_ genes were identified in the context of insertion sequences and transposases, where IS*26* and Tn*4401* (a/b/d isoforms) surrounded *bla*_KPC_, and IS*5*, IS*630*, IS*Ab125*, and Tn*3*-like element IS*3000* surrounded *bla*_NDM_. Several genomes (n = 169; 30%) carried genes associated with three or more classes of antibiotics, characterizing them as MDR, where most of these belonged to ST93 (n = 43), ST4 (n = 14) and ST116 (n = 11) (Table S1). In contrast, there are also genomes of pandemic clones ST4 (n = 39) and ST93 (n = 71) that lack acquired resistance genes (Table S1). Likewise, the Brazilian genomes sequenced here also did not show acquired resistance genes, including the ST93 (Ka-04RR), although this strain is MDR. Only 12 genomes were predicted to harbor the *mcr* gene (Ka37751, GCA_007632255.1; Ka.44724, GCA_007558485.1; Ka.42825, GCA_007558675.1; Ec.42059, GCA_007558345.1; Ka.37756, GCA_007558625.1; Ka.42442, GCA_007558645.1; Ka.43407, GCA_007558665.1; Ka.44253, GCA_007571405.1; Ka.46782, GCA_007558425.1; E845, GCA_023040435.1; 201414107U, GCA_025071235.1; 4417, GCA_024604175.1), where nine were related to ST56 in China (Table S1). Moreover, analyzing PmrA, three Dutch ST364 genomes (isolate 25, GCA_024074185.1; isolate 26, GCA_024074205.1; isolate 27, GCA_024073885.1) were observed carrying the G53S amino acid substitution (Table S2), which has been associated with colistin resistance^9^. The GCA_000950145.1 genome (strain GCSL-DIFS-311) showed a large block of amino acid replacements from position 85 to 109, including insertions and deletions (Table S2), which could also affect its role. Interestingly, the L162M amino acid replacement was observed in almost all genomes (551/561), even in some colistin-susceptible strains (Table S2). Furthermore, three genomes had nonsense mutations in MgrB (N1431, GCA_021837555.1; N676, GCA_021837885.1; CK-00624, GCA_022016415.1), also being associated with colistin resistance^20^.

In vitro analyses showed that Ka-04RR was resistant to meropenem and to most cephalosporins and β-lactams tested (Table 2). However, this strain lacked acquired ESBL and carbapenemases genes (Table S1). Thus, we explored amino acid replacements at chromosomal loci associated with β-lactam resistance in this strain, in addition to the entire genomic dataset. We focused on AmpC and outer membrane porins (Omp35 and Omp36), including their associated regulatory proteins (AmpD, AmpG, AmpR, and OmpR). Comparisons were evaluated using wild-type sequences from carbapenem-susceptible strains. All respective genes (*amp*C, *omp*35, *omp*36, *amp*D, *amp*G, *amp*R, and *omp*R) were identified in most genomes (Table S3). They showed different patterns of single nucleotide polymorphisms (SNPs), some leading to premature translation stops in their respective proteins. The AmpD protein was truncated in 44 genomes, including Ka-04RR. Mutation analysis, considering all genomes, showed that 65% (120/187 sites) of amino acid sites were conserved and several replacements were common, such as V134A, V135A, Q138R (Table S4). Furthermore, replacements associated with carbapenem resistance (P39S, A94T, W95L, S112L, I113S, I160V, R161H^4^) were observed in several genomes, most of them belonging to ST4 and ST93 (Table S4). The AmpG sequences showed 91% (451/491 sites) of conserved amino acid sites (Table S5), where four genomes had truncated proteins, including the Ka01-RR genome, while the Ka02-RR genome lacked *amp*G. Also, several mutational patterns were observed, such as L89I, I93V, T424I, L430V, G461A and I479V (Table S5). Even so, all known AmpG activation motif residues (G25, A122, Q124, A181^21^) were conserved, except for GCA_021937655.1 (strain 122,288) (A181V). AmpR was found truncated in two genomes (presenting premature stop codons in their *amp*R genes) and absent in four genomes (strain UCI 45, GCA_000534135.1; GCSL-DIFS-311, GCA_000950145.1; 035, GCA_011604725.1; NY1688, GCA_022759585.1), which also lacked *amp*C (Table S3). Overall, the genomes showed 87% (255/292 sites) of conserved amino acid sites (Table S6). AmpR sites associated with its role (R86, G102, D135^22,23^ were also conserved except for D135A/N substitutions in four genomes (strain 2485STDY5438452, GCA_900558125.1; 20174220 M, GCA_025115605.1; CK-00624, GCA_022016415.1; 95009, GCA_021936135.1) (Table S6). Regarding the porins, Omp35 was truncated in 21 genomes, but well conserved in the remaining genomes (93% of amino acid sites conserved; 337/359 sites) (Table S7). Most ST202, ST296, ST300, ST375 genomes had common mutations: S182V, D225N, D259N, H260Y (Table S7). Omp36 was the analyzed protein with the highest rates of nonsense mutations, where 98 genomes had premature stop codons. Among the Omp36 positive genomes, several sites had mutations, which resulted in 72% (270/375 sites) of conserved sites (Table S8). Most mutations were concentrated in the final half of the protein (Table S8). Some replacements were frequently found in ST-specific genomes, such as I59V in ST4, ST231, ST237; D189G in ST4, ST16, ST93; D205E in ST93 and ST228 (Table S8). The OmpR showed the highest conservation among the proteins explored with 97% (233/239 sites) of conserved amino acid sites (Table S9). Two important sites (G63 and R150^24,25^) were conserved in all genomes.

Among the proteins analyzed, amino acid replacements and/or premature translation stops in AmpD, AmpR, Omp35, and Omp36, which could lead to a phenotype of greater resistance to β-lactam, were present in 148 genomes spread across the phylogeny, regardless the ST (Fig. 1). Most of these genomes (117/148; ~ 80%) did not contain ESBL (19 of these 148 genomes had ESBL) and/or carbapenemase (12 of these 148 genomes had carbapenemase) genes, suggesting that these mutations could be selection mechanisms in the absence of enzymatic resistance genes. Even more so because most of these genomes (100/148; ~ 67%) also lacked acquired resistance genes (Table S1). Thus, contrasting to the low prevalence of acquired genes, a large proportion of genomes carrying chromosomal alterations associated with resistance to β-lactam was identified.

Integrons (n = 139) could be identified in 96 genomes, mainly from ST93 (n = 29) (Table S10), where almost all were class 1 and only one class 3 integrons (strain 24A19CPO031, DAJACA010000050.1). As integrons are known to capture resistance genes, we searched for these genes in the identified integrons (Table S10). Different combinations of gene cassettes related to resistance were present, where most integrons presented the *dfr*A gene, while a smaller proportion presented *qac*E, *ant*, *aac*, *aph*, *arr*, *bla*_OXA_, and *bla*_GES_, some of them presenting several alleles. Furthermore, *bla*_VIM_ and *bla*_IMP_ carbapenemase genes were also present in gene cassettes (Table S10). Based on the gene cassette sequences of the integrons, 97 of them could be grouped into 22 clusters, with several of them present in genomes of different STs (Table S10).

### *K. aerogenes* virulence analysis

Among the virulence factors identified in the genus *Klebsiella*, the yersiniabactin, colibactin, and aerobactin loci represent clinically important features. Thus, considering the 561 *K aerogenes* genomes, 222 genomes co-carried the *ybt* and *clb* loci, and 16 carried the *ybt* loci, while only one genome presented the aerobactin loci (strain 2022LN-00016, GCA_023553355.1) (Table S1). Furthermore, among the major STs, these loci were prevalent in the genomes of pandemic clones ST93 (n = 114) and ST4 (n = 47) (Table 3). Almost all *ybt* and *clb* loci were in the context of ICEKp10 (208/238 *ybt*+ genomes), regardless of ST, with the majority of ST4 having the *ybt* type 17 (n = 45), and majority of ST93 having the *ybt* type 17 (n = 59) and *ybt* type 20 (n = 52) (Table S1), while only the *clb* type 3 was found associated with these loci. ICEKp other than ICEKp10 were identified in a few genomes and carried only the *ybt*, lacking *clb* (Table S1). Only one environmental genome (ST93) presented the *ybt* 17 and *clb* 3 loci in the context of ICEKp10, while other animal and environmental genomes lacked these virulent loci. Curiously, the salmochelin (*iro*) virulence trait is present in almost all *K. aerogenes* genomes (517/561; ~ 92%) (Table S1), of which 88 did not present the entire cluster (*iro*BCDEN). In addition, although lacking the *ybt* and *clb* loci, most animal and environmental genomes carried the *iro* loci (19/27; ~ 70%). Another prevalent virulence factor among the genomes was the Type VI Secretion System (T6SS), present in 553/561 (~ 98%). Regarding toxins, two factors (EAST1 and *sen*B) were found in the genomes of ST240, ST15, ST93, ST331, ST364. In short, ICEKp10 carrying the *ybt* and *clb* is widespread in different STs, prevailing in ST4 and ST93.

**Table 3:** Prevalence of virulence loci in the main STs of *K. aerogenes.*

ST	#	*ybt*	*clb*	*iro*	T6SS
93	121	114 (94%)	114 (94%)	121 (100%)	118 (97%)
4	57	47 (82%)	47 (82%)	57 (100%)	55 (96%)
135	16	0	0	16 (100%)	16 (100%)
116	13	1 (7%)	1 (7%)	13 (100%)	13 (100%)
197	12	8 (66%)	8 (66%)	12 (100%)	12 (100%)
56	10	0	0	10 (100%)	10 (100%)

In four genomes (GCA_020982565.1, GCA_001631645.1, GCA_003952125.1, GCA_021902315.1), the *ybt* type 4 was predicted, this type being associated with plasmid origin (Table S1). However, analyzing these genomes, we were unable to identify the plasmid replication genes. This could be due to genome assembly fragmentation, however, the *ybt* loci of these genomes were close to integrases and flanked by tRNA–Asn (in 3/4 genomes), which is a feature associated with chromosomal insertion. Thus, if *ybt* type 4 is on a mobile element, it appears to have the ability to integrate into the chromosome.

The US clinical genome GCA_023553355.1 (ST432) was the only one presenting the aerobactin loci (*iuc* 1; *iuc*A-D and *iut*A), in addition to the hypermucoviscosity-associated gene *rmp*A2 (Table S1). These loci likely lie within a genomic island (contig ABGJKZ010000015.1), in a region of approximately 75 kb length (7374 bp–82,380 bp) flanked by repetitive regions (93 bp repeats with 98% identity) and three transposase genes (ISL3-like element, ISNCY-like element). Furthermore, this region has 99% identity and 90% coverage with several *Klebsiella pneumoniae* plasmids (e.g. pFQ61_ST383_NDM-5, pAP855, pVir-CR-hvKP-C789), some of them designated as hypervirulent plasmids. Curiously, although only this genome contained the *iuc*A-D loci, most genomes (n = 552; 98%) harbored the *iut*A gene, which encodes the aerobactin receptor.

### *K. aerogenes* plasmid resistome/virulome

We explored the 561 genomes for potential plasmids based on plasmid replicons, and identity and coverage with previously reported plasmids. Contig sequences presenting plasmid replicons and with high identity and coverage to previously reported complete plasmids were found in 317 genomes (317/561; 56%), which totaled 541 putative plasmid sequences (Table S11). Although it is not possible to state whether these contigs represent entire plasmids or fragments thereof, the presence of the plasmid replicon associated with high identity and coverage to previously reported plasmids suggests that these contigs belong to elements with an extra-chromosomal nature. The putative 541 plasmids had a median size of 10 kb and 52% GC content; ranging from 1 to 6 per genome (median of one plasmid); and ColRNAI was the most common replicon type. Based on the mobility markers present in these putative plasmids, we predicted that 51 would be conjugative (44.9 kb median size, 47% GC content), as they carried relaxase, *vir*D4, and *vir*B4 homologues genes^26^; 339 would be mobilizable (9.4 kb median size, 55% GC content) due to the presence of oriT sequences and/or relaxase genes, without T4SS-like genes; and 151 would be non-mobilizable (16 kb median size, 49% GC content) (Table S11). These putative plasmids were mainly harbored in the ST93 (n = 93) and ST4 (n = 34) genomes but were also present in other STs in smaller numbers (n < 10). These genomes are from several continents, presenting a wide time scale (2002 to 2021).

**Figure 2:**
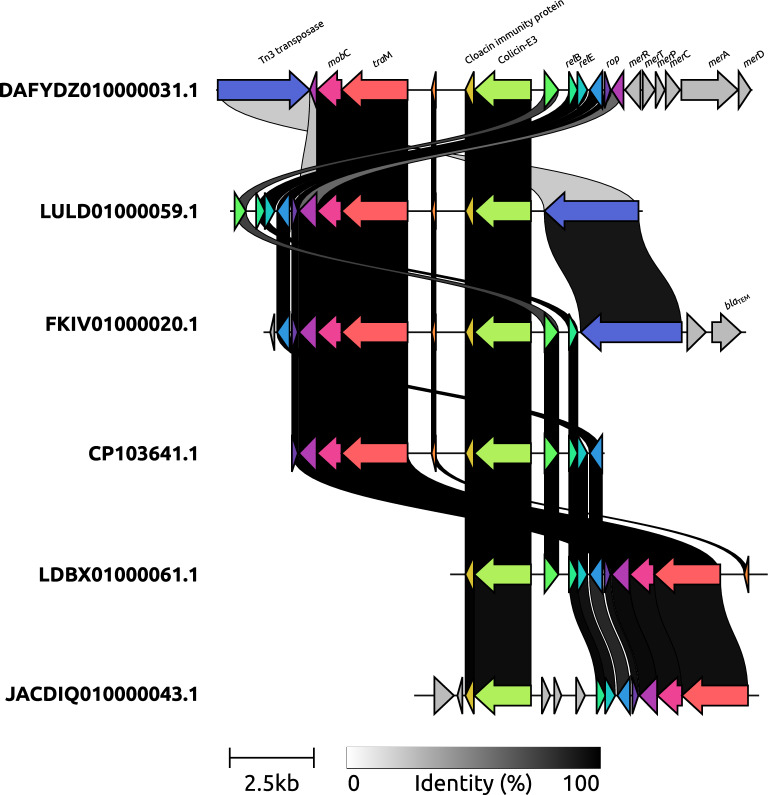
Synteny variations of putative *K. aerogenes* ColRNAI plasmids. A comparison of putative plasmids is shown, where the cloacin gene and its immunity gene, *tra*M, *mob*C, *rel*B/*rel*E toxin/antitoxin, and *rop* genes appear to represent a core segment.

Interestingly, most of the putative plasmids carrying the ColRNAI replicon (172/194; ~ 88%) had a median length of 9.2 kb and a similar structure, mainly comprising a cloacin gene (a type of colicin) and its immunity gene, *tra*M, *mob*C, *rel*B/*rel*E toxin/antitoxin, and *rop* genes (Fig. 2). Variations were observed in these mobilizable sequences, for example, the sequence DAFYDZ010000031.1 presented genes related to mercury resistance; FKIV01000020.1 presented the *bla*_TEM_ gene; and Tn3 transposase was present in sequence LULD01000059.1 (Fig. 2). Virulence genes were rare in the putative plasmids, where only five had one gene, including the *tra*J (associated with invasion), EAST1 (exotoxin), and *sen*B (exotoxin) genes (Table S12).

Considering resistance genes, 111/541 putative plasmids (in 82 genomes), with a median size of 46.1 kb, carried 1 to 12 genes (median of two genes) (Table S13), associated with several classes of antibiotics, including cephalosporins (*bla*_TEM_, *bla*_CTX-M_, *bla*_OXA_), carbapenems (*bla*_NDM_, *bla*_IMP_, *bla*_KPC_, *bla*_VIM_), aminoglycosides (*aac*, *aad*, *ant*, *arm*A, *aph*A), phenicol (*cat*), sulfonamides (*sul*), macrolides (*mph*A, *msr*E), quinolones (*qnr*S). The most prevalent resistance genes in the putative plasmids were BRP (gene encoding bleomycin resistance protein) (present in 22 putative plasmids); *bla*_NDM-1_ (n = 17); *bla*_TEM-1_ (n = 16); *bla*_KPC-2_ (n = 12); *qnr*S1 (n = 12), where the majority were spread across genomes of different STs (Table S13). Importantly, the *mcr* gene (colistin resistance) was associated with putative plasmids (10/12 genomes with *mcr*). These genomes belonged to ST56/China (8/10; IncI2 replicons) and ST207/Singapore (2/10; IncX4 replicons), in which the Chinese ones co-harbored the *bla*_CTX-M-199_ gene (Table S13). Moreover, most of these plasmids were predicted as conjugative (Table S13). Interestingly, these plasmids had approximately 100% identity and coverage with various *E. coli* plasmids (e.g. pC6-2, pEC1188-MCR, pZE36, pMCR-1_Msc, pDIB-1, pMCR_1139_A1) from strains from various locations around the world, which suggests that these elements are subject to horizontal transmission. In addition, the *bla*_NDM-1_ gene was carried in 17 putative plasmids of 11/24 positive *bla*_NDM-1_ genomes, mainly in the ST197/Singapore genomes (featuring two plasmids, each carrying a copy of *bla*_NDM-1_) that also co-harbored the BRP gene (Table S13). Interestingly, some integrons (n = 14) were present in plasmids, in which CP042532 plasmid harbored the *bla*_IMP_, *aac*, *cat*B, *qac* cassette genes; and CP070519 plasmid harbored *qac*, *arr*, *cat*B, *aac*, *bla*_OXA-1_ cassette genes.

Although most genomes are of human origin, few putative plasmids from other sources could be observed, some of them carrying resistance genes (e.g. CP050069 from water, carrying *aph*, *mph*A, *qnr*, *bla*_VIM_, *cat*, *dfr*A, *sul* genes; CP047668 from chicken manure, carrying *aph*, *bla*_CTX-M-65_, *fos*A, *bla*_NDM_ genes).

## Discussion

There is a historical gap in the epidemiology of *K. aerogenes*, as it has always been considered an opportunistic pathogen, but recently interest in this organism has increased, mainly due to the emergence of carbapenem-resistant isolates^3^. Furthermore, this species was recently renamed, being previously known as *Enterobacter aerogenes*, which still influences researchers to keep the old species name, even in recent publications. However, in genomic information databases such as GenBank, this has been updated. Until now, studies focused on this emerging pathogen were small-scale, considering few genomes and/or with a restricted geographic perspective. Here, in addition to generating new genomes of this species in Brazil and contextualizing them in the global scenario, we carried out an in-depth analysis of the resistome, virulome, and plasmidome in view of the emergence of *K. aerogenes* as a pathogen.

Previous genomic epidemiological analyzes have shown that *K. aerogenes* ST4 and ST93 have been implicated as the dominant global clones^4^, as have occurred in a few countries in the Americas, Europe, and China. In fact, here, expanding this analysis, encompassing all *K. aerogenes* genomes, these STs continue to represent the dominant clones, causing infections/outbreaks in several other countries (e.g. Belgium, Germany, Japan, Qatar, Singapore, South Korea, Switzerland, Thailand, United Kingdom), thus characterizing them as pandemic clones that impact the clinics.

*K. aerogenes* genomes can be clustered by the presence/absence of two virulence determinants: the yersiniabactin and colibactin^5^. In fact, it is observed that most STs lack these loci, while they are predominant in the ST4 and ST93 genomes. These virulence loci were found in several ICEKp, mainly in ICEKp10, and their distribution is not homogeneous among all genomes, even within the same ST, which shows their mobile characteristic. In *K aerogenes*, ICEKp10 had already been identified in a few strains of ST4 and ST93 from the USA^4^. The present analysis revealed a wide distribution of this virulence-associated element within this species, particularly in ST4 and ST93 genomes from several countries around the world. Moreover, colibactin, a genotoxin that can induce DNA damage in eukaryotic cells and tumor formation^27^, raises concerns about its high prevalence and distribution in *Klebsiella* species.

In contrast to the *ybt* and *clb* loci, the salmochelin loci (*iro*), another virulence trait identified, was present in a high proportion of genomes (~ 92%), including those of animal and environmental origin. This result is similar to the findings of other studies considering smaller genome sets^5,28^, and suggests that *K. aerogenes* may be a potential reservoir of virulence genes for other bacteria^28^. In fact, in *K. pneumoniae*, salmochelin is detected in low prevalence, being more identified in mobile elements, such as plasmids^29^. Although prevalent, 88 *K aerogenes* genomes had incomplete salmochelin loci. In avian pathogenic *Escherichia coli*, it was observed that the absence of *iro*C, *iro*DE, or *iro*N abrogated the virulence of the bacteria^30^. Thus, it can be hypothesized that the *iro* loci in these *K. aerogenes* would not act on virulence as in *E. coli*. Furthermore, the presence of incomplete loci may also suggest that other functions could be involved, such as colonization and survival linked to commensalism, as they can increase the fitness of strains within a specific niche.

A putative virulence island, carrying the aerobactin loci (*iuc*A-D and *iut*A) and *rmp*A2 gene, was found in a *K. aerogenes* genome (GCA_023553355.1). This island has also been observed in several *K. pneumoniae* plasmids. In fact, recently, a transposon harboring the aerobactin operon was identified in *K. pneumoniae* virulence plasmids^31^. Most of these plasmids harbor the *rmp*A/*rmp*A2 genes, aerobactin, and salmochelin loci, which greatly enhance the virulence of *K. pneumoniae* strains^32^. Although salmochelin is strongly associated with aerobactin in *K. pneumoniae*^33^, this was not observed in *K. aerogenes* as *iro* was prevalent and *iuc* rare. Thus, we show here that a mobile element carrying *K. pneumoniae* hypervirulence marker genes is being transferred between species of the genus *Klebsiella*.

Although most genomes did not carry acquired antibiotic resistance genes, having only the intrinsic resistome, this does not mean an absence of resistance, since in vitro analyses of Ka-04RR showed its MDR profile, and further in silico analyses revealed the presence of alterations in chromosomally encoded factors associated with this MDR profile. Indeed, chromosomal alterations have been observed in several other genomes. Most genomes (74%; 411/561) do not carry carbapenemase genes (including Ka-04RR), however, almost all of them carry the *amp*C gene, which if overexpressed could lead to carbapenem resistance^3^. In fact, AmpC is chromosomally encoded by *K. aerogenes,* and alterations in its regulatory genes could affect AmpC translation^21^. Here we observed several amino acid replacements in AmpC regulatory proteins, some of them already associated with resistance to carbapenems (mainly in AmpD), in addition to a large set of other replacements yet to be characterized in further studies. Moreover, ~ 21% of the genomes showed truncated Omp35 and Omp36 porins, which could also increase resistance to β-lactam agents^3,34^. Therefore, regardless of the absence of β-lactamase genes in most genomes, we raised evidence of other possible mechanisms that could confer some level of resistance to β-lactams in *K. aerogenes* circulating the world. Unlike *K. pneumoniae*, reports of integrons in *K. aerogenes* are scarce^35–37^. Here, this genetic element was identified in ~ 17% of the analyzed genomes, presenting several combinations of gene cassettes, some not yet reported in this species, such as *bla*_OXA-1_, *bla*_OXA-10_, *bla*_IMP_, *bla*_VIM_, *bla*_GES_*.* Thus, carbapenemase genes can also be captured by these genetic platforms in this species.

In Brazil, studies on *K. aerogenes* have focused on molecular analyses of resistance genes from carbapenem-resistant isolates. These studies cover isolates from three Brazilian regions (Minas Gerais, Paraná, and Pernambuco states), most of them co-harboring the *bla*_KPC-2_ and *bla*_TEM_ genes^38–40^. In fact, we observed that several Brazilian of ST4 and ST93 genomes presented these genes (Table S1), and plasmids carrying carbapenemase genes (*bla*_KPC-2_, *bla*_NDM-1_) have already been observed among *K. aerogenes* in the country^41,42^. In addition, we provided new genomes from other regions, expanding regional epidemiology. Although data on resistance genes in *K. aerogenes* in Brazil are available, most studies have not defined the ST of these strains, with only reports of ST93 and ST16^10,43^. Thus, the current epidemiological scenario of *K. aerogenes* in Brazil is mainly driven by ST93, ST16 and also by ST4, as is the case worldwide. In addition, a clinical *bla*_NDM_-producing *K. aerogenes* was recently identified in the country belonging to ST128^44^. Here, ST128 was also associated with environmental (Asia) and animal (Africa) strains, therefore revealing the One Health trait of this species, particularly of ST128. Furthermore, this ST is widespread and evolving in the context of the acquisition of resistance and virulence genes.

In China, *bla*_NDM_ alleles were associated with plasmids in *K. aerogenes* ST4^45,46^. Here, the *bla*_NDM_ gene was also found in several putative plasmids from Singaporean genomes. Interestingly, both countries were the only ones to present genomes with putative plasmids carrying the *mcr* gene (colistin resistance). Indeed, the initial report of *mcr* in plasmids occurred in China (2016)^47^, and our analysis showed that the presence of the *mcr* gene in putative plasmids of *K. aerogenes* still seems to be restricted to Asia. Even though these putative plasmids are already widespread in *E. coli* worldwide. Although plasmids drive the exchange of antibiotic resistance in other countries, we could only observe five Brazilian ST93 genomes harboring putative plasmids with *bla*_KPC-2_. Therefore, other transfer mechanisms may be acting in other acquired genes. Nevertheless, if plasmids are also driving *K. aerogenes* adaptation in Brazil, more sequences should be made available to determine this question, in addition to more data for epidemiology.

The most common type of plasmid replicon identified was the ColRNAI, as observed by Passarelli–Araujoa et al.^5^ on a smaller set of genomes. Interestingly, 172/194 of ColRNAI replicon putative plasmids have been observed carrying colicin E3 (cloacin-like; rRNase activity) and its immunity gene. Colicins are bacteriocins produced by some gram-negative bacteria, showing antibacterial activity against closely related species, and being highly found in natural populations of *E. coli*^48,49^. Indeed, blasting cloacin-bearing *K. aerogenes* putative plasmids, several *E. coli* and *K. pneumoniae* plasmids showed high coverage and identity of > 75% and > 99%, respectively. It has been reported that Cloacin-like exhibits a weaker effect and narrower spectrum of activity against several species, but exhibits high inhibitory activity against *K. aerogenes*, which suggests that these plasmids are associated with the ecology of this species^49^. Interestingly, most *K. aerogenes* genomes contained the *iut*A gene, which encodes a receptor for aerobactin, a rare virulence factor in the current dataset. However, curiously, this receptor is also used by cloacins^49^, suggesting that the main function of this receptor in *K. aerogenes* may not be related to virulence, but perhaps to ecological functions.

In conclusion, *K. aerogenes* is a species capable of acquiring resistance genes from other organisms, but it seems to be more prone to becoming resistant by mutations in housekeeping genes, something that could only be assessed by in-depth analysis since most of the in silico resistome survey platforms are not up to date regarding this information. Furthermore, there are *K. aerogenes* lineages circulating around the world carrying virulence genes that may be determinants for its emergence as a clinical pathogen.

## Methods

### Public data set

All reported *Klebsiella aerogenes* genomes (n = 557), including complete and draft ones, were obtained from the Genome database of the National Center for Biotechnology Information (NCBI) in October 2022. The accession numbers are supplied in Table S1.

### Isolates and genome sequencing and assembly

In this study we analyzed four *Klebsiella aerogenes* strains from nosocomial cases in the Amazonic and Southeast regions of Brazil: Ka-01RR (subclavian vein catheter tip), Ka-02RR (tracheal secretion), Ka-04RR (unknown), Ka-06RJ (sputum). The genomic DNA extraction was done using the NucleoSpin Microbial DNA kit (Macherey–Nagel), and the genomic libraries were constructed using Nextera paired-end library. The sequencing was performed using Illumina Hiseq 2500, generating reads of 150 bp length. The raw reads were filtered and trimmed using NGS QC Toolkit v.2.3.3^50^ with a Phred score ≥ 20. The genomes were de novo assembled using SPAdes assembler v3.14.1^51^.

### Antimicrobial susceptibility testing

The four *Klebsiella aerogenes* strains were grown on Mueller Hinton Agar at a temperature of 35–37 °C for antimicrobial susceptibility testing by the disk-diffusion method using 24 antibiotics (amikacin, gentamicin, streptomycin, tobramycin, neomycin, kanamycin, imipenem, meropenem, ertapenem, ceftazidime, ceftriaxone, cefotaxime, cefepime, cefoxitin, cefuroxime, ampicillin/sulbactam, piperacillin/tazobactam, ticarcillin/clavulanic acid, ciprofloxacin, levofloxacin, trimethoprim/sulfamethoxazole, minocycline, tetracycline, colistin) considered for *Enterobacteriaceae* resistance classification^19^ and interpreted according to the Clinical and Laboratory Standards Institute (31st ed.)^52^. The minimum inhibitory concentration of colistin was determined by the broth microdilution method and interpreted according to the European Committee for Antimicrobial Susceptibility Testing (EUCAST) guidelines (MIC breakpoint for resistance > 2 mg/L)^53^.

### Genome characterization

The *K. aerogenes* genomes were submitted to the Kleborate v2.1.0^33^ pipeline to sequence typing, and identification of acquired virulence and antibiotic resistance genes. Integrons were survey with Integron Finder^54^. Type VI Secretion System (T6SS) was identified using the T6SS prediction tool^55^. Insertion Sequences and transposases associated with antibiotic resistance genes were identified using TnFinder^56^.

Plasmid identification was based on the characterization of contigs carrying a plasmid replicon marker (*rep*), using PlasmidFinder database (v2.1.1)^57^, and presenting identity (=> 70%) and coverage (=> 50%) to known plasmids, using the PLSDB (v.2023_11_03_v2), a curated database of complete bacterial plasmids^58^. The putative plasmids were further characterized for virulence and antibiotic resistance genes using ABRicate v1.0.1 (https://github.com/tseemann/abricate) with the VFDB (accessed in Jun/2023)^59^ and CARD (v3.2.7)^60^ databases, respectively (identity and coverage => 60%). Plasmid clustering was done using CD-HIT-EST at 90% identity and 70% coverage^61^. The mobility of the putative plasmids was predicted based on the presence of gene markers, such as relaxase, Type IV Secretion System (T4SS) genes (e.g. VirB4 and VirD4), and OriT sequences (origin sites of DNA transfer), as described^26,62^. The putative plasmids were classified as: conjugative, if encoding relaxase, *vir*B4, and *vir*D4 homologues; mobilizable, if encoding relaxase and/or oriT sequences, and lacking the T4SS; and non-mobilizable, if not encoding relaxase and/or oriT sequences. These searches were performed using hmm profiles as previous described^62^, in addition to the *vir*B4 homologue CagE_TrbE_VirB (Pfam PF03135), present in some *Proteobacteria*.

### Phylogeny

The genomes were annotated by Prokka v1.12^63^. *K. aerogenes* core genome was estimated using Roary v3.13 with default parameters (considering a minimum percentage identity of 95%)^64^, and single nucleotide polymorphisms (SNPs) were extracted from the concatenated core genes with snp-sites v2.5.1^65^. Next, phylogenetic analysis was performed by IQTree v1.6.12^66^ using the GTR+ F+ ASC+ R5 model to obtain a maximum likelihood tree with 1000 ultrafast bootstrap replicates^67^. The tree was designed using iTOL platform^68^.

### Supplementary Information

Supplementary Table S1.

Supplementary Table S2.

Supplementary Table S3.

Supplementary Table S4.

Supplementary Table S5.

Supplementary Table S6.

Supplementary Table S7.

Supplementary Table S8.

Supplementary Table S9.

Supplementary Table S10.

Supplementary Table S11.

Supplementary Table S12.

Supplementary Table S13.

## Data Availability

The data analyzed in this study is available in the supplementary information files. The genomes generated are available under BioProject PRJNA1000993 and GenBank accession numbers: JAUUCT000000000 (Ka-01RR), JAUUCS000000000 (Ka-02RR), JAUUCR000000000 (Ka-04RR), JAUUCQ000000000 (Ka-06RJ).
